# Research on demands and accessibility of health services for AIDS long-surviving patients with AIDS-nonrelated diseases: a survey in central China

**DOI:** 10.7448/IAS.17.4.19728

**Published:** 2014-11-02

**Authors:** Na He, Yingfeng Ye

**Affiliations:** School of Public Health, Fudan University, Shanghai, China

## Abstract

**Introduction:**

Compared with western countries, China started to provide free medicine for AIDS patients years later, which leads to the late emergence of problems on health service demands of AIDS long-surviving patients with non-AIDS-related diseases. Government hasn't laid enough stress on it.

**Materials and Methods:**

The interviews and questionnaire surveys are conducted and analyzed to get information. The interviewees include 81 AIDS long-surviving patients in three villages and several hospitals in Shangcai, Zhumadian, and 18 AIDS-related decision makers and health service providers.

**Results:**

There are 79 long-surviving patients out of 81. 58 patients have non-AIDS-related diseases. 21 patients get hypertension and 28 get HCV. 100% patients have been to the clinics with their real-name IC cards for minor illness. 43 patients have been transferred to assigned hospitals at the county level. Seven have the experience utilizing health services in the municipal or provincial assigned hospitals. The problem is on accessibility. 40 patients hope to get more convenient and cheap health services. Among them, 37 say the kinds and the amount of medicine in village clinics are not adequate. Seven give up because of the expensive treatment expense. For 21 patients with hypertension, 3 buy medicine at the county-level hospitals. The other 18 choose to buy at private pharmacy. For 28 patients with HCV, 3 are not aware they actually got HCV. Free hepatic protector medicine is provided at village clinics. Up to 11 patients have not taken any treatment for HCV.

**Conclusions:**

Patients with hypertension go to the private pharmacy for medicine instead of higher level hospital because of lack of medicine in clinics, far distance from hospitals, cumbersome procedures in hospitals, limited dosage of prescriptions and too little discount. The situation for patients with HCV is even worse. It is predicted 70% of AIDS long-surviving patients have HCV. The treatment is expensive and out of pocket. And free liver-protection medicine does not work sometimes. Some patients working outside their home town do not want to reveal their health situation to get free medicine. The elderly with multiple co-morbidities need more caring. Government should expand the scale of free medicine. Hospitals need to improve medicine plans and assist on medicine purchase. For patients, attitude decides everything.

